# Hyperspectral Analysis of Soil Nitrogen, Carbon, Carbonate, and Organic Matter Using Regression Trees

**DOI:** 10.3390/s120810639

**Published:** 2012-08-03

**Authors:** Stephan Gmur, Daniel Vogt, Darlene Zabowski, L. Monika Moskal

**Affiliations:** School of Environmental and Forest Sciences, College of the Environment, University of Washington, Seattle, WA 98195, USA; E-Mails: dvogt@uw.edu (D.V.); zabow@uw.edu (D.Z.); lmmoskal@uw.edu (L.M.M.)

**Keywords:** soil horizons, Washington, Oregon, ASD

## Abstract

The characterization of soil attributes using hyperspectral sensors has revealed patterns in soil spectra that are known to respond to mineral composition, organic matter, soil moisture and particle size distribution. Soil samples from different soil horizons of replicated soil series from sites located within Washington and Oregon were analyzed with the FieldSpec Spectroradiometer to measure their spectral signatures across the electromagnetic range of 400 to 1,000 nm. Similarity rankings of individual soil samples reveal differences between replicate series as well as samples within the same replicate series. Using classification and regression tree statistical methods, regression trees were fitted to each spectral response using concentrations of nitrogen, carbon, carbonate and organic matter as the response variables. Statistics resulting from fitted trees were: nitrogen R^2^ 0.91 (*p* < 0.01) at 403, 470, 687, and 846 nm spectral band widths, carbonate R^2^ 0.95 (*p* < 0.01) at 531 and 898 nm band widths, total carbon R^2^ 0.93 (*p* < 0.01) at 400, 409, 441 and 907 nm band widths, and organic matter R^2^ 0.98 (*p* < 0.01) at 300, 400, 441, 832 and 907 nm band widths. Use of the 400 to 1,000 nm electromagnetic range utilizing regression trees provided a powerful, rapid and inexpensive method for assessing nitrogen, carbon, carbonate and organic matter for upper soil horizons in a nondestructive method.

## 1. Introduction

Although soils are often considered as just thin layers of surficial unconsolidated material, they are a vital component of an interconnected ecosystem that influences every landscape. For example, the variability of soil properties across a landscape can influence habitat types which then shape the distribution of different animal species. It has even been suggested that as a fundamental land resource, soil productivity has influenced the economy and development of many countries and, hence, “the advancement of the modern world” [1]. But when the soils are degraded, such as through poor agricultural practices, it has been shown that entire civilizations can collapse [2]. Today, knowing the importance of our soils, we place value on monitoring them for any changing soil conditions (e.g., soil degradation). It is therefore essential that there are effective and sensitive tools developed to monitor and evaluate soil properties in order to better understand their potential effects on productivity. Traditional soil analysis techniques require time intensive methods which become limiting when applied at regional or global scales [3]. Therefore, the development of alternative tools to inexpensively, rapidly and accurately evaluate the spatial variability of soils is needed to enable informed policies and land-use decisions.

It has been demonstrated that due to the spatial variability of soils, creating an accurate and spatially explicit representation of soils within an area can be cost prohibitive [4]. Remote sensing technologies using varying reflectance spectroscopy methods with satellite, aerial and laboratory settings have been increasingly explored in alternative methods. These new methods have been used to decrease costs while trying to maintain or even increase accuracy and spatial resolution so that they can better identify and characterize “physical, chemical, and biological properties” of soils [5]. Some examples of successful, multi-scaled, utilizations of multispectral and hyperspectral sensors range from mapping of salt-affected soils using Landsat [6], to using a satellite platform to model soil heat flux using airborne hyperspectral sensors over farmlands [7], measuring tropical soil characteristics using narrow band hyperspectral models [8] in a laboratory setting or country level mapping of soils using 2,350 samples from across Australia [9]. These applications highlight the diversity of possible uses and have led to the identification of different soil properties and types through nondestructive methods. The synergy from these results has been enhanced by the creation of spectral libraries of the different soils and their specific characteristics at varying spatial extents. These spectral libraries now allow other researchers to explore their own data and statistically analyze them for unique patterns associated with the spectral frequencies and soils and their properties.

These spectral libraries are a compilation of soil reflectances, or the amount of measured electromagnetic energies, that have been reflected from the surface of the soils. The reflections are mostly related to the inorganic solids, organic matter, air and water of the soils [10] and the various combinations of those soil components change as soil development or formation occurs. Examples of some factors that most commonly affect the soils and soil properties (*s*) as described by V. V. Dokuchaev in Russia and others such as by H. Jenny in the U.S. are climate (*cl*), organisms (*o*), topography (*r*), parent material (*p*) and time (*t*) [11]. Integrating these factors to express the dynamic nature of soil formation has been shown in the following equation provided by Hans Jenny:

s=f(cl,o,r,p,t)

This equation puts forth the idea that for any specific soil property within a soil “such as pH, clay content, porosity, density, carbonates, *etc.*” [12] that property is a function of soil forming factors, each being independent but working in unison to form unique soils. By monitoring any changes of these soils or their soil properties allows us to better determine the soil's health or potentially enhance our soil management activities. This is where remote sensing technologies using reflectance spectroscopy may be used to aid our monitoring of soil conditions. Thus by measuring the unique spectral signature of a soil sample, characteristics of that soil sample may be modeled from chemical laboratory reference measurements by using multivariate statistical methods to give us a more informed understanding of an *in situ* soil property or soil. These reference soil samples would have been characterized using traditional chemical analytical methods and then, coupled with the laboratory-derived spectral measurements, correlations between soil spectra and specific soil properties could be explored.

Energy emitted from the surface of a soil has been measured by a substantial number of researchers in applications that have developed a diverse spectral library of African soils, linking remote sensing information to spatial prediction of soil functional capacity [5] and developing on-the-go utilizing Visible to Near Infrared (VISNIR) soil spectroscopy to estimate soil organic carbon and clay concentration [12] or pH [13] at the field level. Beyond the wide application of VISNIR remote sensing for the determination of different soil properties, the electromagnetic ranges considered optimal for the determination of different properties has varied. Using a spectral range of 1,300 to 2,500 nm, Chang *et al.* found a high correlation between predicted values of total C, N, moisture and other soil properties [14]. Krishman *et al.* utilized the visible part of the electromagnetic spectrum using bands 0.6236 and 0.5644 µm to predict organic matter and obtained a maximum R^2^ value of 0.98 which they found better than using the Near Infrared (NIR) region which yielded a maximum R^2^ of 0.87 [15]. Classification and regression trees (CART) within R have been used for ecological applications including, for example, relating soil properties with lead levels [16], explaining woody cover influenced by precipitation in Africa [17], modeling of forest productivity using remote sensing [18], and other ecological modeling which linear regressions fail to fully characterize [19].

Addressing a need to quantify landscape-level soil productivity, this study expanded upon previous research by utilizing new remote sensing technologies and statistical models. Observation of terrestrial conditions such as soil properties allows for the indexing of potential below-ground productivity, carbon sequestration or other applications through the analysis of soil spectra. Within this context sampling of soils within Washington and Oregon was conducted to establish a correlation between analyzed soil spectra and their specific soil properties. Characterization of soil samples were analyzed using traditional laboratory analytical methods and also spectrally analyzed in the electromagnetic range of 400 to 1,000 nm. The following questions were examined for this study:

- What are the variations of soil spectra? Is there variation within the soil samples that allows differentiation between different soil series or within series?
- What effect does different concentrations of nitrogen, carbon, carbonate and organic matter have on the spectral signature of the soil samples?
- Can regression trees be used to model the carbon, nitrogen, organic matter and carbonate concentrations from soil spectra and chemical laboratory reference measurements?

## 2. Experimental Section

**Figure 1.**
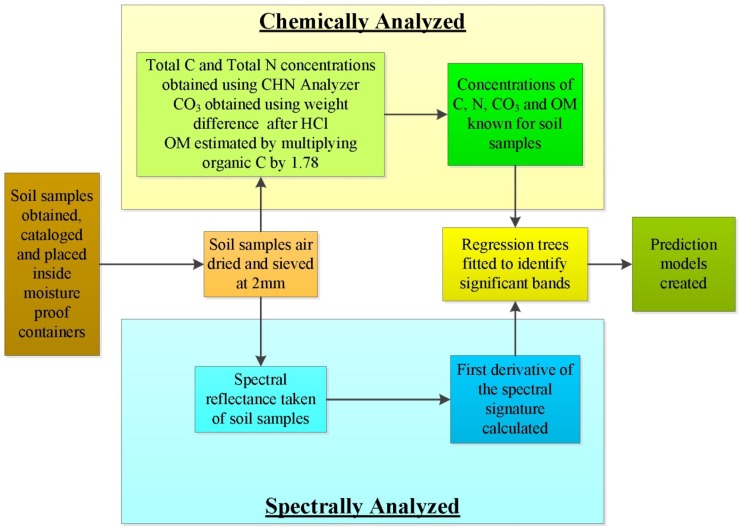
Generalized workflow used in this study showing steps taken to chemically and spectrally analyze the soil samples and then create models to predict the concentrations of the soil's total carbon, total nitrogen, carbonate carbon and organic matter.

Using selected soil samples obtained from locations within Washington and Oregon, the methodology outlined in the following sections was used to create prediction models for concentrations of total nitrogen, total carbon, carbonate carbon and organic matter. A generalized workflow is outlined in Figure 1.

### 2.1. Selected Soil Samples

Thirty-nine archived soil samples of horizons from profiles of different soil orders were used for spectral analysis. These soils had been previously analyzed for total nitrogen, carbon, and carbonate concentrations [20,21]. Data from these chemical analyses were then correlated with spectral analyses obtained in this study. Three replicate soil profiles were sampled from each soil series. Soil samples were acquired from a soil pit dug to the deepest depth possible allowed by site conditions or 2-m maximum. Soil samples of volumes up to 3,000 cm^3^ were collected for laboratory analysis from the horizons of each soil pit. Soils were then air dried and sieved for <2 mm fine-soil fractions. After laboratory analyses, residual samples were stored in Ziploc bags and archived in boxes until retrieved in the summer of 2010 for spectral analysis for this study. Spectral analyses for this study were conducted on a subset of the stored samples which were chosen to represent the greatest difference across the soil orders sampled within Washington and Oregon. Descriptions of the soils chosen to build this spectral library are presented in Table 1.

The concentration of total nitrogen, total carbon, carbonate carbon and organic matter within the selected soil samples are displayed within Table 2. The concentrations of each soil property were obtained using traditional soil testing methods then these values were correlated to the measured spectral response obtained from each soil sample.

**Figure 2.**
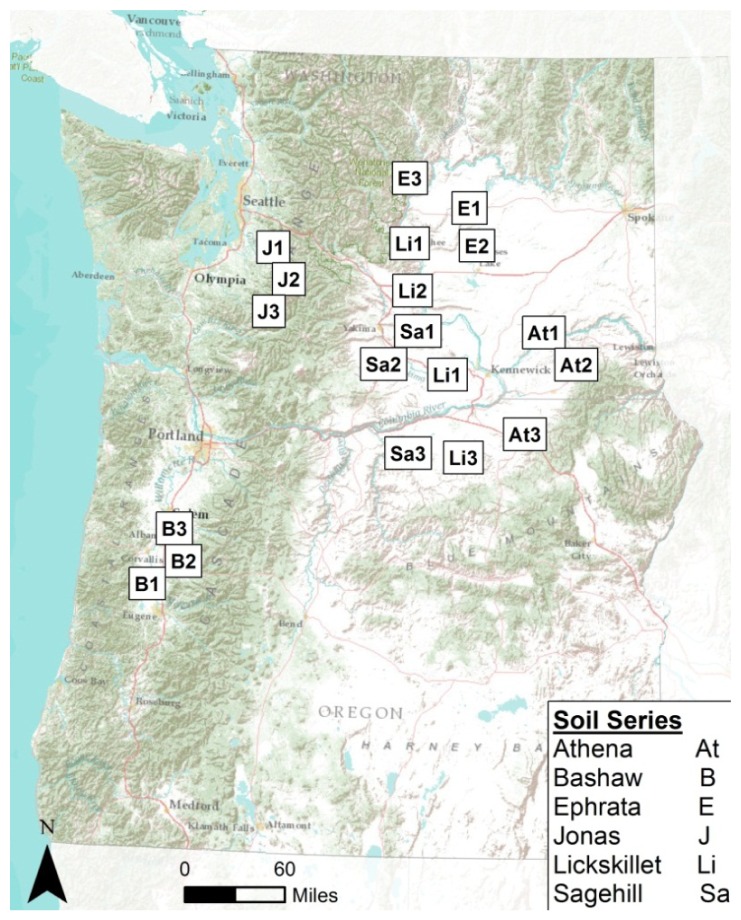
Sites across Washington and Oregon highlighting the approximate locations of where soils were obtained for this study.

Spatial distribution of the soil samples across Washington and Oregon are shown in Figure 2. The spatial extent of selected samples were confined to Washington and Oregon to allow a diverse sample set while minimizing the geographic range which has been shown to affect the accuracy of organic carbon predictions [22].

### 2.2. Spectroradiometer

Reflected light from the surface of the soil samples was measured using a FieldSpec Handheld portable spectroradiometer from Analytical Spectral Devices, Inc. (ASD, Boulder CO, USA). The device measures the VISNIR spectrum, the 325–1,075 nm electromagnetic range that is sampled with a 512-channel silicon photodiode array that is overlaid with an order separation filter. Each channel has a width of 1.6 nm with a dedicated detector and a spectral resolution of about 3 nm at approximately 700 nm [23]. Documentation for the FieldSpec spectroradiometer describes the field of view for the device as having a 10:1 ratio for distances from target to the aperture size of the optical fiber exposed in the front of the device providing about a 3-cm sampling area across the soil surface at a distance of 0.3 m. Spectral information is collected from the FieldSpec spectroradiometer through a serial cable connection connected to a laptop running ASD's software package FieldSpec R3.

### 2.3. Data Acquisition

Soil spectra were obtained in a laboratory with staging configured to reduce the amount of scattered light. Each soil sample was placed on a stage platform approximately 0.3 meters from the ASD FieldSpec spectroradiometer and illuminated from above using two tungsten quartz halogen filament lamps containing 250 W bulbs and aluminum reflectors (Lowel Pro-light, Lowel-Light Manufacturer Inc., New York, NY, USA); these bulbs produce a ∼3,200 K color temperature (Ushio GCA, Cypress, CA, USA) similar to the apparatus used by Shepard *et al.* [5]. The lamps were placed on each side of the stage with the light beams directed at a 20 degree angle from vertical and elevated about 1 m from the sample location. The stage setup was fabricated using heavy-weight black poster board spray-painted with a flat matt black and a table with a surface area of 0.58 m^2^. Three sides of the stage were surrounded with the poster board 0.91-m high to block any reflected light from the surrounding white walls in the laboratory.

Each soil sample was filled to the top of a 14-cm diameter × 1-cm high tin which was painted flat matt-black; the soil sample surface was smoothed to remove variation from the surface that would introduce variation to the spectral signature among the replicate soil samples. Each soil sample was scanned ten times by being placed under the spectroradiometer which was calibrated by first taking a dark current reading using the internal shutter then using a white spectralon disk to take a white reading. This process was repeated three times for each of the soil samples which were scanned singularly in succession for a single run, and then this process was repeated 3 times. In total 30 scans of each soil sample were obtained over the entire time the lab setup was in operation ensuring that if any variations occurred to the light sources or the spectroradiometer, these variations would be captured and averaged over all the readings.

### 2.4. Laboratory Processing

The percent total nitrogen and carbon of each soil sample were determined using a PerkinElmer 2400 CHN analyzer (PerkinElmer Corp., Norwalk, CT, USA) after sieving each sample to <2 mm then grinding with a mortar and pestle [20,21]. To derive concentration of carbonate carbon for each soil sample, the method of weight difference was used following treatment with HCl [21]. Organic matter for each soil sample was estimated by subtracting percent inorganic carbon from the percent total carbon then multiplying that resultant by 1.78 based on the assumption that the organic matter contains about 56% carbon [24].

### 2.5. Spectral Data Processing

The mean reflectance from each run was visually compared by overlaying each series of scans to ensure similar results for each individual sample, confirming that no human or electronic errors were encountered during data collection. The mean reflectance of the three runs created a spectral signature of each soil sample and those values were exported to an ASCII format which was then imported into spreadsheet software and R [25] for further analysis. Using the library rpart within R, a statistical analysis was conducted for carbonate, carbon, nitrogen and organic matter using a regression tree model of the samples gathered. The usable electromagnetic range of the Fieldspec spectroradiometer in this study was determined to be 400 to 584 and 632 to 1,000 nm with the range of 585 to 631 nm discarded due to fluctuations.

### 2.6. Spectral Analysis

Soil spectral signatures were tested for similarity between and within soil orders. This process used ENVI (ITT Visual Information Solutions, Boulder, CO, USA) Spectral Analyst which created a ranked or weighted score based on the input of the spectral information. These scores were rated for similarity of soils within the selected sample set in an effort to determine the separation possibility of each spectral signature. The underlying analysis used Binary Encoding, Spectral Angle Mapper and Spectral Feature Fitting to create a similarity score to compare to all other soil samples within the spectral library [26]. The ranked similarity scores created range from 0 to 1, in which 1 is an absolute match and 0 would imply no similarities. For example, a comparison score of 0.98 between spectra would indicate very similar spectral analyses. In contrast, a score of 0.50 would indicate some similar spectral properties but different enough in other aspects [26]. The similarity weighting score created is a resultant summary of equal weighting of the comparison methods Binary Encoding [27], Spectral Angle Mapper and Spectral Feature Fitting. Binary Encoding classification technique encodes the data into a binary representation (1 or 0) if a band falls above or below the spectrum mean thus returning a score based on the number of bands that match the reference spectra. Spectral Angel Mapper is a physically-based spectral classification through an n-D angle in a pairwise comparison method, once a vector of angles is calculated then a spectral similarity score is returned. Spectral Feature Fitting takes the reference spectra and compares the other spectra using a least squares technique. Before the analysis process, each method was given an equal weight of 0.333, and then the analysis was begun with each method assessing the pairwise similarity using the appropriate algorithms. Once the analysis had been completed, a final score was assembled from the resultant summary of each weighted method.

### 2.7. Fitting Regression Trees

The first derivative of each soil's spectral signature was used to create a regression tree to generate a predictive model which minimized error without ‘over fitting’ the data. Use of the first derivative of the spectral signature is a common practice with statistical models using reflectance of the electromagnetic spectrum which eliminates the albedo effect of the data while highlighting the instantaneous slope between bands [5,14]. The regression tree is built by taking the sample population and finding the best variable which divides the single group into two new groups. This process is then applied again, treating each new group as its own unique entity and finding the next variables which best divide up those two groups into four. The process is carried out continually or recursively until a minimum size is reached or a subgroup can no longer be subdivided [28]. The result is a tree like structure to represent the recursive partition. Each node or leaf represents a portion of the original population and has a simple mathematical model which applies to that node. To ensure that a regression is not ‘over fitted’ to the data, the relative error is minimized. Trees with a relative error, which is calculated 1-R^2^, close to 0 produces a good prediction while a relative error around, or greater than, one produces a poorer prediction [29]. Once the lowest relative error has been chosen, a complexity parameter is selected which minimizes the cross-validation prediction error which may increase as additional splits are introduced to the fitted tree. The cross-validation error is calculated through a leave one sample out technique for estimating a generalization error based on “resampling” to test subsequent sub-trees from the full tree. This value is expressed within the RPART library using the *printcp* command which will print a table showing the unique complexity parameter, the number of splits and the associated cross-validation error [30]. The complexity parameter is the measure of cost for adding additional variables to the model. The best complexity parameter reflects a tree which the highest number of significant factors which result in a low complexity parameter score [28]. Each regression tree was cross-validated 1,000 times to ensure replication in trees fit for all response variables. Classification and regression trees are non-parametric with no assumptions made about the underlying distribution of the predictor variables [31,32].

## 3. Results and Discussion

### 3.1. Soil Order/Series Comparison

Resulting similarity scores for the soil samples were diverse, allowing for the spectral separability between replicate soil series and horizons. A selected number of similarity scores can be seen in Table 3.

Overall trends of similarity scores between soil samples are not consistent for either within or between soil series. Soils may have different spectral signatures for a variety of reasons. For example, they may have differing percentages of sand, silt and clay (*i.e.*, soil texture) or because they may be composed of different mineralogies such as biotite mica which is darker in color or the lighter colored calcites such as found in sedimentary carbonates like limestone. Soil spectra may also be influenced by other materials such as organic matter or even soil moisture. In Table 3 the soils with the lowest similarity scores were Ephrata 1 Ck and Ephrata 3 Ck which are highlighted in blue and green and then followed by SageHill 1 Bk1 and SageHill 1 Bk4 highlighted in orange and brown. The lower similarity scores for soil samples Ephrata 1 Ck and Ephrata 3 Ck might be attributed to carbonates or limestone fragments within the horizon [33]. Overall the distributions of similarity scores for all other samples have a tighter distribution range between 0.80 and 0.98 (Table 3). Within the scope of this study, the similarity scores of the selected soil samples were diverse, allowing separation between individual soil samples. The tool of spectral similarity has been used in many fields, such as geology, to relate an unknown sample to known spectral signatures of collected samples. A similar process could be used to identify soils or describe their nutrient properties by comparing their spectral signatures to other libraries. Other soil spectral libraries that could be utilized include the USGS digital spectral library [34] which contains mostly mineral references, or the World Agroforestry Center (ICRAF) and ISRIC World Soil Information database [35].

### 3.2. Spectral Library

**Figure 3.**
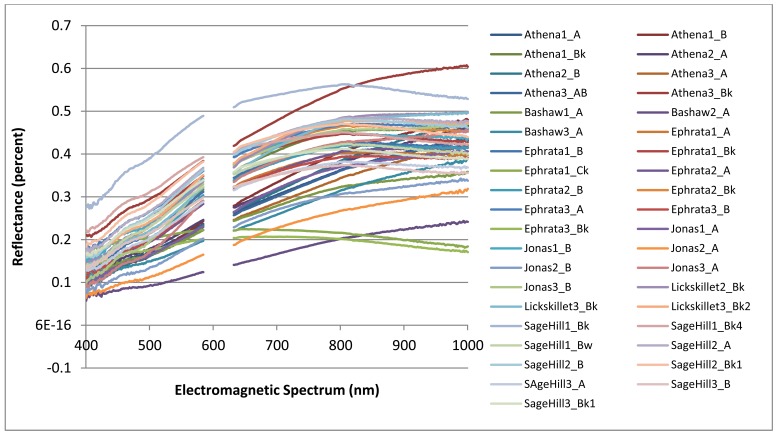
Complete soil electromagnetic spectrum derived from an ASD HandHeld FieldSpec spectroradiometer and the spectral band width regions used within the spectral analysis of the selected soils.

**Figure 4.**
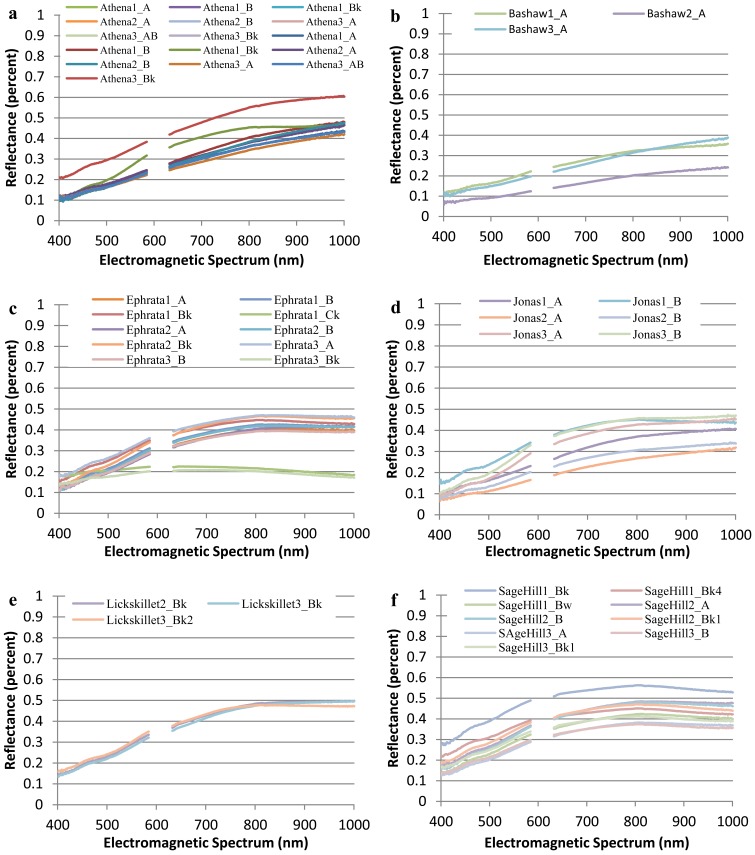
Plots of the spectral signatures by individual soil series: (**a**) Athena, (**b**) Bashaw, (**c**) Ephrata, (**d**) Jonas, (**e**) Lickskillet, and (**f**) SageHill.

The collected spectral signatures, shown in Figures 3 and 4, represent a spectral library that can be used in future applications by others who wish to integrate a library of soil spectra into their study. Collection of soil spectra within a confined laboratory setting allowed for the consistant and systematic acquisition of spectral signatures under controlled lighting conditions as reflected by previous studies [5,14]. As opposed to obtaining soil spectra in a field setting where illumination is frequently inconsistant between different soil pit locations as has been observed in other studies [36,37].

Figure 4 which highlights the range of soil spectra within series while Figure 3 highlights the overall variablity of spectra of all soils selected for this study. In Figure 3, the spectral signatures for Ephrata 1 Ck and Ephrata 3 Ck have a flatter slope with a much lower percent reflectance than the other spectral signatures. The overall shape of each spectral signature appears to be a result of its varying parent material and different soil properties.

These spectral libraries could be enhanced by linking known soil properties measured using traditional chemical analytical methods in the laboratory with existing spectral library signatures obtained from soils at the same locations. Efforts to create informational databases of soil through the construction of spectral libraries have centered on the viewpoint of linking remote sensing to measuring unique soil characteristics to ascertain soil functional capacity, such as net primary productivity [5]. Continued development of spectral libraries has been undertaken with the goal of linking satellite remote-sensing technologies to applications of soil mapping. Continued technological improvements of sensors on satellites provide a large potential for enabling landscape-level management decisions and policies using information about resources and allocation for restoration or preventative actions [38].

### 3.3. Predictive Models

Resulting regression trees for the soil properties of total nitrogen and carbon, carbonate, and organic matter created predictive models which correlated the laboratory obtained results with the spectral signature of each soil sample. A summary of the R-squared value for each model, its associated errors and the significant spectral bands at each step of the recursive process is presented in Table 4.

**Figure 5.**
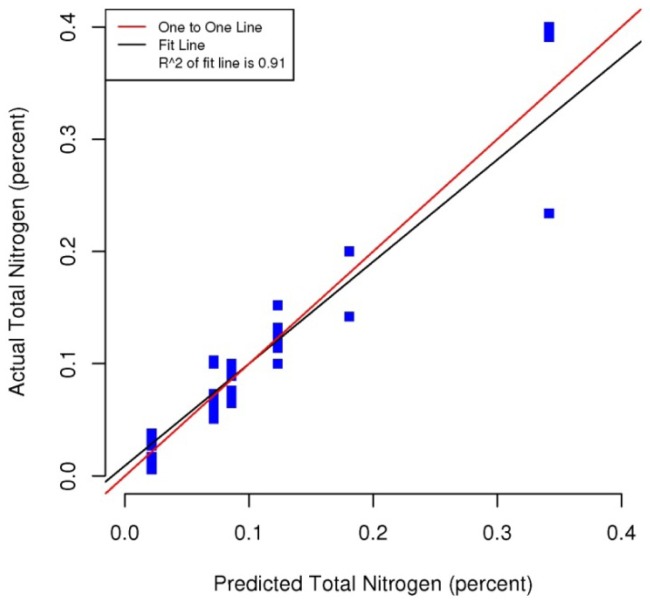
Scatterplot and fitted line of actual values and predicted values from the regression tree for percent total nitrogen of the soil samples (R^2^ = 0.91, *p* < 0.01, n = 38).

**Figure 6.**
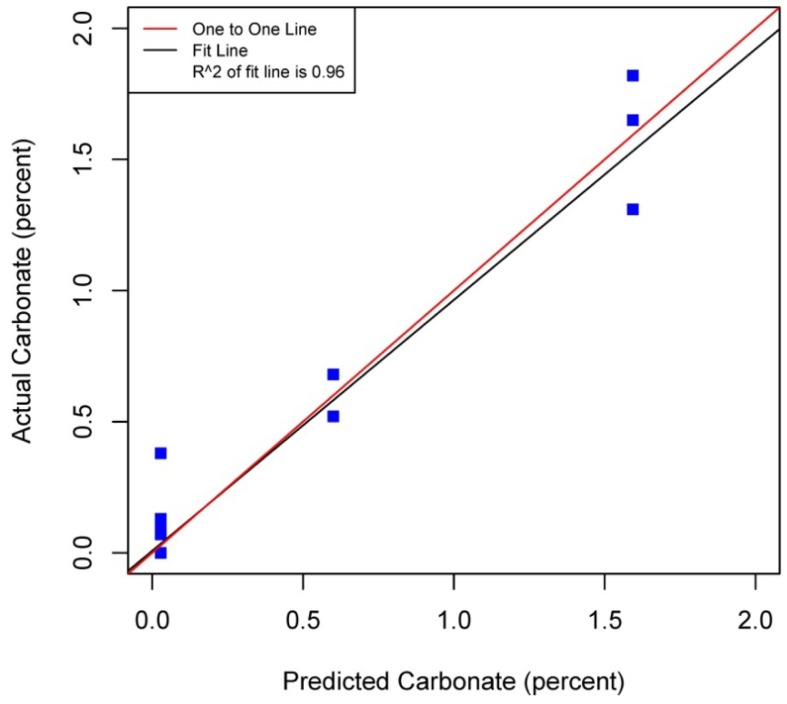
Scatterplot and fitted line of actual values and predicted values from the regression tree for percent carbonate of the soil samples (R^2^ = 0.96 *p* < 0.01, n = 38).

**Figure 7.**
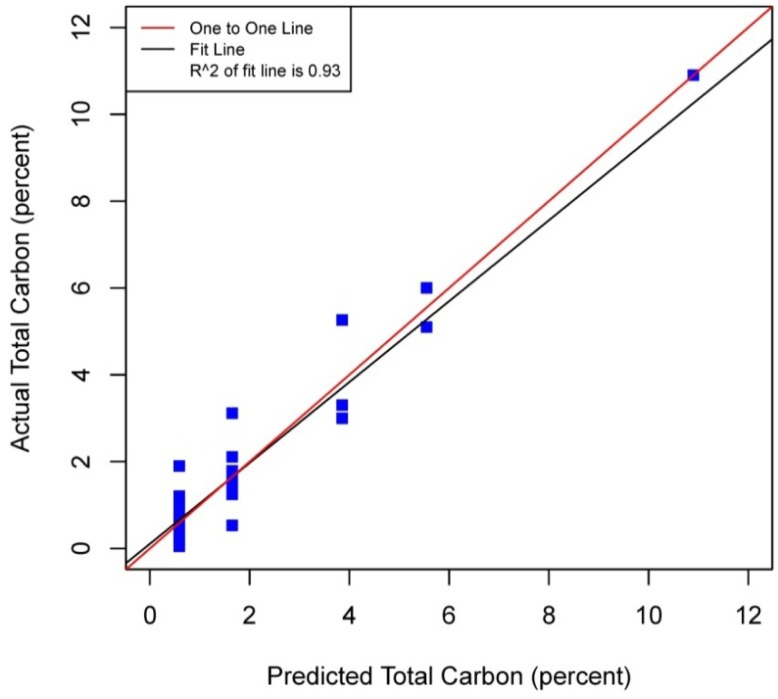
Scatterplot and fitted line of actual values and predicted values from the regression tree for percent total carbon (organic carbon plus carbonate carbon) of the soil samples (R^2^ = 0.93 *p* < 0.01, n = 38).

**Figure 8.**
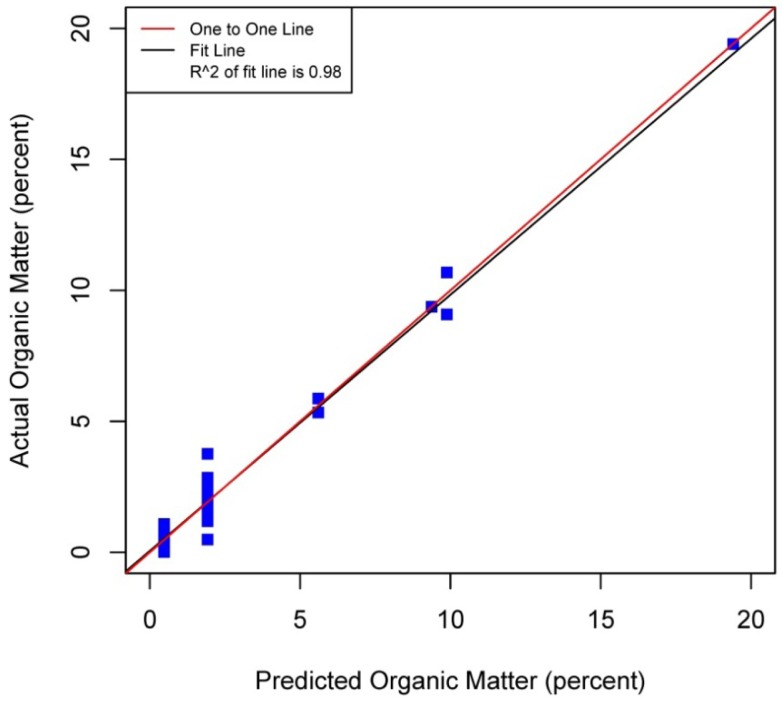
Scatterplot and fitted line of actual values and predicted values from the regression tree for percent organic matter of the soil samples (R^2^ = 0.98 *p* < 0.01, n = 38).

Figures 5–8 plot the predicted concentrations of nitrogen, carbon, organic matter, and carbonate for each soil sample using spectral analysis verses the actual amount obtained from traditional chemical analytical methods.

Using the soil samples gathered for this study, regression trees were fitted to the spectral signatures collected to correlate measured soil properties of carbonate, nitrogen, carbon and organic matter. Studies using a similar electromagnetic range had results with R^2^ values of 0.86 for organic matter [39] using the neural network statistical method. Additional models for total carbon with an R^2^ of 0.87 for total carbon using principal component analysis [14], and 0.91 by using partial least squares regression [40]. Three different studies looking at organic carbon created prediction models of R^2^ = 0.79 using principal component regression [41], 0.80 using multivariate adaptive regression splines [5] and 0.89 using partial least squares regression [36]. Two other studies looking at total nitrogen resulted in prediction models with R^2^ values of 0.85 using principal component regression [14] and 0.86 using partial least squares regression [39]. Previous studies have not utilized CART to identify statistically significant bands for soil carbon, nitrogen and organic matter. The prediction models created using CART for soils in this study are equal to or slightly better than previous studies. When looking at the quality of the model selections for the fitted regression tree in this study, nitrogen and carbonate have the lowest cross-validation prediction errors. The greater the cross-validation error, the greater the likelihood that a tree will ‘over fit’ the data which will provide a predication tree that will give unreliable extreme predictions [30]. However the low cross-validation error for carbonate may be misleading due to a clustering of the carbonate values. All soil samples were included in this model, even those soils without carbonate. This created a strong bias towards zero. That coupled with 7 of the 13 soils containing carbonate concentrations of less than 0.1 percent and the remaining carbonate samples ranged from greater than 0.1 percent to 1.6 percent carbonate, all have created some clustering of data. Future enhancements of the spectral signature library for the soils sampled from the Washington/Oregon area would increase soil samples that have carbonate values that more fully complete the range of potential carbonate values to give more confidence in any estimated R^2^ values associated with a predictive model. The prediction model for nitrogen which had a low cross-validation error and unique grouping created through the leaf node configuration using significant bands was a better fit then previous studies which used other statistical methods. The resulting CART model using soil samples from Washington and Oregon the spectral reflectance explains 91 percent of the variability (*p* < 0.01) in soil nitrogen. The next step to utilizing these prediction models is to expand the geographic area or acquire additional samples from the existing study area. A subset of the new samples would be tested using traditional chemical laboratory techniques to obtain percent nitrogen, carbon, and carbonate then the results could validate the existing prediction model. Percent nitrogen, carbon, carbonate and organic matter can be inferred from the spectral reflectance off the surface of the soil using the prediction model.

## 4. Conclusions

Assessment of the selected sample set of soils acquired in Washington and Oregon using spectral analysis first established that these representative samples were spectrally separable using the electromagnetic range of 400 to 1,000 nm. Variations across the samples and within soil series created unique spectral signatures which were correlated with soil properties: total carbon, inorganic carbon, carbonate and organic matter. Regression trees fitted to the first derivative of the spectral signature yielded predictive models which showed promising results for further applications of correlating soil properties to the spectral response. Use of remote sensing technology to measure soil spectra provides an alternative method which correlates a specific spectral response with soil properties. Previous studies have mostly focused on the use of the electromagnetic spectrum range between 1,000 nm and 2,500 nm, creating high-probability prediction models developed from relatively time-intensive regression model techniques and higher cost sensors. In this study by analyzing soils from Washington and Oregon and using this more narrow spectral range in partnership with highly powerful classification and regression tree method, high probability regression models were created that are comparable to previous studies that have been undertaken for soils found in other regions around the world. The results of this study yielded prediction models for total nitrogen with an R^2^ of 0.91 at 403, 470, 687, and 846 nm band widths, 0.95 R^2^ for carbon carbonate at 531 and 898 nm band widths, 0.93 R^2^ for total carbon at 400, 409, 441 and 907 nm band widths, and 0.98 R^2^ for organic matter at 300, 400, 441, 832 and 907 nm band widths. The models for nitrogen, total carbon and organic matter provided better fits to predicted soil quantities than previous studies. The spectral reflectance used to create prediction models through building regression trees identified significant bands. Locations of splits within the regression trees were diverse, often first occurring at the lower end of the electromagnetic range used then next at a higher band in the range. The significant bands identified do not represent a single linear equation that can be used as a prediction model but a diverse set of equations at each leaf node to create a regression tree. The complexity of the regression trees created closely fit the training soil reflectances used within this study, outperforming previous studies which used this electromagnetic range and other studies which used a wider electromagnetic range. Also this study's prediction model for carbonate had only three samples of soil carbonate concentrations greater than one percent, thus providing a limited range for the prediction model. Therefore this carbonate model should not be used outside the range of carbonate concentrations found in the soils from this study. Utilization of the methodologies presented in this study can be extended into the spatial mapping of soil carbon, nitrogen and organic matter across other Washington and Oregon areas through the collection and spectral analyses of additional samples from the field or even capturing remotely sensed data from aerial hyperspectral platforms or the Hyperion satellite sensor from areas where the soils are exposed [42,43].

Use of a laboratory-based soil spectra collection method allowed for the conformation that soil spectra were separable and specific spectral bands could be associated with total nitrogen, total carbon, organic matter and carbon carbonate. Extension of these analyses methods into a field setting encounter new obstacles including variability of light source [44], variation on soil moisture [37] and other factors which can be controlled within a laboratory setting. Techniques used in recent studies employ air drying of field samples then acquiring the spectral reading [45] using a contact probe device which contains its own light source [41,45]. Use of a contact probe removes the reliance on the sun's illumination while approximating the light source used within a laboratory setting. Studies which use these techniques and technologies have pointed to a reduction in the variation between field-obtained soil spectral and laboratory-based collection methods [41].

## Figures and Tables

**Table 1.** Soil series names, USDA soil classifications, soil horizons and their parent materials of selected soil samples from Washington and Oregon used in this study.

**Soil Series**	**Subgroup Classification**	**Soil Order**	**Horizons**	**Parent Material**

Athena	Pachic Haploxeroll	Mollisol	A, AB, Bw, Bk	loess mixed with volcanic ash
Bashaw	Xeric Endoaquert	Vertisol	A	alluvium from igneous rock
Ephrata	Xeric Haplocambid	Aridisol	A, Bw, Bk, Ck	glacial outwash mixed with loess
Jonas	Typic Hapludand	Andisol	A, B	residuum/colluvium from andesite, an admixture of volcanic ash and pumice
Lickskillet	Lithic Haploxeroll	Mollisol	Bk, Bk2	colluvium mixed with loess, rock weathered from basalt or rhyolite
Sagehill	Xeric Haplocalcid	Aridisol	A, B, Bk, Bk1, Bk4, Bw	lacustrine deposits with a mantle of loess or eolian deposits

**Table 2.** The laboratory-derived concentrations of total nitrogen, total carbon, carbonate carbon and organic matter from the selected soil samples used within this study [20,21].

**Soil Sample**	**Concentrations**			

	**Total Carbon** **(Percent)**	**Total Nitrogen** **(Percent)**	**Carbonate Carbon** **(Percent)**	**Organic Matter** **(Percent)**

Athena 1 Ap	1.41	0.11	0.00	2.51
Athena 1 Bw	0.70	0.07	0.00	1.25
Athena 1 Bk	0.27	0.05	0.10	0.29
Athena 2 Ap	2.11	0.15	0.00	3.76
Athena 2 Bw	0.77	0.09	0.00	1.37
Athena 3 Ap	1.21	0.12	0.00	2.15
Athena 3 AB	0.98	0.10	0.00	1.74
Athena 3 Bk	1.05	0.07	0.38	1.19
Bashaw 1 Ap	1.25	0.09	0.00	2.23
Bashaw 2 Ap	5.27	0.39	0.00	9.37
Bashaw 3 Ap	3.30	0.23	0.00	5.88
Ephrata 1 Ap	0.54	0.10	0.00	0.95
Ephrata 1 Bw	0.27	0.10	0.00	0.49
Ephrata 1 Bk	0.39	0.07	0.13	0.45
Ephrata 1 Ck	0.08	0.03	0.07	0.01
Ephrata 2 Ap	1.00	0.12	0.00	1.78
Ephrata 2 Bw	0.40	0.07	0.00	0.72
Ephrata 2 Bk	0.37	0.07	0.11	0.45
Ephrata 3 A	0.61	0.07	0.00	1.08
Ephrata 3 Bw	0.32	0.06	0.00	0.57
Ephrata 3 Ck	0.05	0.03	0.05	0.09
Jonas 1 A	5.10	0.20	0.00	9.08
Jonas 1 Bw	1.40	0.10	0.00	2.49
Jonas 2 A	10.90	0.40	0.00	19.40
Jonas 2 Bw	6.00	0.20	0.00	10.68
Jonas 3 A	3.00	0.10	0.00	5.34
Jonas 3 Bw	1.60	0.10	0.00	2.85
Lickskillet 2 Bk	0.87	0.13	0.07	1.42
Lickskillet 3 Bk	3.12	0.14	1.82	2.31
Lickskillet 3 Bk (2)	1.79	0.08	1.31	0.85
SageHill 1 Bk	1.90	0.03	1.65	0.45
SageHill 1 Bk (4)	0.55	0.01	0.52	0.06
SageHill 1 Bw	0.40	0.06	0.00	0.71
SageHill 2 A	0.72	0.05	0.00	1.29
SageHill 2 Bw	0.40	0.04	0.00	0.72
SageHill 2 Bk (1)	0.86	0.03	0.68	0.32
SageHill 3 A	0.29	0.02	0.00	0.51
SageHill 3 Bw	0.14	0.01	0.00	0.25
SageHill 3 Bk (1)	0.13	0.01	0.10	0.05

**Table 3.** Selected similarity scores of some of the different soil horizons evaluated in this study, generated by ENVI spectral analyst using Binary Encoding, Spectral Angle Mapper and Spectral Feature Fitting.

	Athena1 A	Athena1 Bw	Bashaw1 A	Ephrata1 A	Ephrata1 Bw	Ephrata1 Ck	Ephrata3 Ck	Jonas1 A	Jonas1 Bw	Lickskillet2 Bk	SageHill1 Bk1	SageHill1 Bk4	SageHill2 A	SageHill2 Bw
Athena1 A	1													
Athena1 Bw	0.93	1												
Bashaw1 A	0.94	0.88	1											
Ephrata1 A	0.87	0.87	0.90	1										
Ephrata1 Bw	0.87	0.86	0.90	0.96	1									
Ephrata1 Ck	0.61	0.50	0.63	0.64	0.66	1								
Ephrata3 Ck	0.65	0.55	0.67	0.69	0.71	0.93	1							
Jonas1 A	0.92	0.88	0.93	0.88	0.88	0.64	0.70	1						
Jonas1 Bw	0.85	0.84	0.88	0.94	0.95	0.70	0.76	0.88	1					
Lickskillet2 Bk	0.90	0.87	0.94	0.94	0.94	0.68	0.73	0.92	0.90	1				
SageHill1 Bk1	0.80	0.69	0.81	0.83	0.85	0.79	0.84	0.80	0.84	0.84	1			
SageHill1 Bk4	0.79	0.69	0.81	0.83	0.85	0.79	0.84	0.79	0.83	0.83	0.98	1		
SageHill2 A	0.88	0.84	0.90	0.95	0.96	0.72	0.77	0.88	0.95	0.93	0.91	0.91	1	
SageHill2 Bw	0.87	0.82	0.90	0.94	0.96	0.72	0.77	0.89	0.94	0.94	0.91	0.91	0.97	1

**Table 4.** Predictive statistics derived using the regression tree method and associated parameters when creating predictive models of percentages of total nitrogen and carbon, carbonate, and organic matter using spectral bands between 400 and 1,000 nm for different soil series and horizons from Washington and Oregon.

**Soil Property**	**R^2^**	**p statistic**	**Relative Error**	**Cross Validation Error**	**Complexity Parameter**	**Spectral Bands (nm)**			

						**First Split**	**Second Split**	**Third Split**	**Fourth Split**

Total Nitrogen	0.91	*p* < 0.01	0.09	0.35	0.01	470	846	403, 687	515
Carbonate	0.96	*p* < 0.01	0.71	0.71	0.17	531	898		
Total Carbon	0.93	*p* < 0.01	0.07	1.37	0.04	441	907, 400	409	
Organic Matter	0.98	*p* < 0.01	0.02	1.16	0.01	441	907, 400	832, 300	
